# Medications for Alcohol Use Disorder Among Patients With Severe Alcohol-Related Liver Disease

**DOI:** 10.1001/jamanetworkopen.2025.59016

**Published:** 2026-02-11

**Authors:** Ram Sundaresh, Jasleen Singh, Julio Meza, Sammy Saab, Akshay Shetty

**Affiliations:** 1 Department of Medicine, University of California, Los Angeles; 2 Department of Medicine, University of Michigan, Ann Arbor; 3 Department of Surgery, University of California, Los Angeles; 4 Department of Family Medicine, University of California, Los Angeles

## Abstract

**Importance:**

Alcohol-related liver disease (ALD) is the leading indication for liver transplant in the US; however, use of medications for alcohol use disorder (MAUDs) remains very low overall. Few studies have examined the use of MAUDs for patients with severe ALD, and lack of clinical knowledge around the benefits of MAUDs for severe ALD remains a barrier to their use.

**Objective:**

To assess the association between use of MAUDs and mortality among patients with severe ALD referred for liver transplant.

**Design, Setting, and Participants:**

This retrospective single-center cohort study was conducted at a tertiary referral center among 1309 patients with severe ALD referred for liver transplant between January 1, 2016, and December 31, 2022. Statistical analysis was performed from January 2023 to December 2025.

**Exposure:**

Use of MAUDs, including US Food and Drug Administration–approved and off-label treatments.

**Main Outcomes and Measures:**

The main outcome was all-cause mortality, as determined by clinical staff and documented in the electronic medical record.

**Results:**

This study included 1309 patients (mean [SD] age, 57.1 [10.5] years; 989 men [75.6%]) and had a mean (SD) of 38 (25) months of follow-up. Use of MAUDs for at least 3 months was associated with 6.6% higher 1-year survival (SE, 0.02%) and 18.5% higher 3-year survival (SE, 0.03%). This association persisted in propensity score–adjusted multivariable Cox proportional hazards regression models, independent of Model for End-Stage Liver Disease score, liver transplant status, and other clinical factors (hazard ratio, 0.59; 95% CI, 0.39-0.92).

**Conclusions and Relevance:**

In this cohort study, use of MAUDs was associated with improved survival among patients with severe ALD. This finding highlights the need for improved access to MAUDs among patients with severe ALD.

## Key Points

**Question:**

Is the use of medications for alcohol use disorder (MAUDs) associated with lower long-term mortality among patients with severe alcohol-related liver disease (ALD)?

**Findings:**

In this cohort study of 1309 adults undergoing liver transplant evaluation, use of MAUDs for at least 3 months was associated with a significant survival benefit, with 6.6% higher 1-year survival and 18.5% higher 3-year survival.

**Meaning:**

This study suggests that, among patients with alcohol use disorder, pharmacotherapy should be considered, even for patients with severe ALD.

## Teaser

This cohort study assesses the association between use of medications for alcohol use disorder (AUD) and mortality among patients with severe alcohol-related liver disease (ALD) referred for liver transplant.

## Introduction

Alcohol use remains prevalent worldwide and in the US, with a significant increase noted during the recent COVID-19 pandemic.^1^ High-risk alcohol use increased by nearly 30% from 2002 to 2012, with a disproportionate increase seen among women and members of racial and ethnic minority groups, while recent survey studies suggest that nearly 30 million people in the US have an alcohol use disorder (AUD).^2,3^ These trends in alcohol use have transformed alcohol-related liver disease (ALD) into the most common indication for hospitalization and mortality among patients with chronic liver disease.^4^ Estimates suggest that approximately 17 million people in the US have some form of ALD, and since the COVID-19 pandemic, there has been a marked increase in ALD mortality, disparities, and onset at a younger age.^5,6^ ALD is the current leading indication for liver transplant in the US and has an important impact on public health at the national level.^7^

A mainstay of treatment for ALD is the avoidance of alcohol intake. For patients with AUD, recommendations for avoiding alcohol may be insufficient to achieve sobriety, especially for those with moderate to severe AUD. Although liver injury in ALD may be reversed with avoidance of alcohol, AUD treatment is far more complex, requiring decoupling of the reward processing associated with alcohol use, and involves several patient-specific factors such as AUD severity, patient insight, physiological dependence, having a support system in place, and concurrent mental health disorders. Effective treatment of ALD and AUD requires a holistic approach to addressing all of these factors to foster sustained recovery.

Multiple treatment options, including psychosocial treatments and medications for AUD (MAUDs), are currently available for patients with AUD. Treatment with MAUDs has been associated with reduction in alcohol use and improvement in AUD outcomes.^8,9,10,11,12^ Prior studies have also shown that MAUDs reduce the incidence and progression of ALD, while also improving survival.^13,14^ However, despite the significant association of AUD and ALD with public health, treatment rates for underlying AUD remain low overall. Recent estimates reported that only 7.8% of US adults with AUD received any treatment in 2023, while only 2.0% of these patients received MAUDs.^15^ In comparison, AUD treatment among patients with liver disease was somewhat higher, ranging from 10% to 14% for any treatment, and use of MAUDs was even lower, at 0.4% to 0.8%.^16,17^

This low rate of AUD treatment has important implications for patients with severe ALD, who face the highest mortality rates among patients with chronic liver disease.^4^ Undertreatment of AUD among this patient population is likely due to multiple factors including stigma, low rates of screening, and clinician barriers such as lack of knowledge around clinical management or benefits of MAUDs.^18,19,20,21^ Few studies have examined the use of MAUDs among patients with severe ALD. The aim of this study is to assess the association between use of MAUDs and mortality among patients with severe ALD who were referred for liver transplantation.

## Methods

### Study Population

A retrospective cohort study was performed to assess the association of MAUDs with mortality among patients with severe ALD. A total of 2567 eligible patients who were referred to a tertiary center for liver transplant evaluation for ALD between January 1, 2016, and December 31, 2022, were identified (eFigure in Supplement 1). Documentation from the transplant committee evaluation includes a committee note that specifies the primary cause of liver disease as determined by the transplant committee. Text parsing of these notes was used to identify patients with ALD as the primary cause of liver disease (eMethods 1 in Supplement 1). This process identified 1309 patients who were referred for liver transplant evaluation and had ALD as their primary cause of liver disease, which defined the cohort for this study. Patients were excluded if they did not have ALD listed as their primary cause of liver disease or if they were younger than 18 years. The study protocol was independently reviewed and formally approved by the University of California, Los Angeles, institutional review board, which waived patient consent because data were deidentified. Study findings are reported in accordance with the Strengthening the Reporting of Observational Studies in Epidemiology (STROBE) reporting guideline.^22^

### Exposure

The primary exposure of interest was use of MAUDs. US Food and Drug Administration–approved treatments for AUD such as naltrexone, acamprosate, and disulfiram were included, along with commonly used off-label treatments for AUD such as gabapentin, baclofen, and topiramate. Electronic medical record (EMR) prescription data were used to identify patients who were taking any of these medications. For survival analyses and multivariable modeling, we set a minimum duration of medication use of at least 3 months of MAUDs, which was the median duration of MAUD use among participants who survived during the study period.

### Outcome and Covariates

The primary outcome of interest was all-cause mortality, which was identified from an EMR-based designation as deceased. This was determined by clinical or administrative staff who were attempting to follow up with the patient and were notified that the patient was deceased. Additional sociodemographic factors were collected from the EMR including age, self-reported race and ethnicity (Asian, Black, Hispanic, White, other race or ethnicity [Indigenous, Middle Eastern or North African, multiple races, or self-identified as “other”], and unknown race or ethnicity), marital status, primary language, zip code of residence, and Area Development Index (a zip code–based indicator of neighborhood deprivation). Race and ethnicity data were collected to assess our study cohort characteristics and to help control for sociodemographic factors within our multivariable regression models. Additional clinical covariates included Model for End-Stage Liver Disease–Sodium (MELD-Na) score (range, 6-40, with higher scores suggesting worse disease severity), liver transplant status, liver decompensations, other liver complications, and Charlson Comorbidity Index. Liver decompensations included bleeding esophageal varices, ascites, and hepatic encephalopathy. Other liver complications included hepatocellular carcinoma, hepatorenal syndrome, hepatopulmonary syndrome, coagulopathy, and portal vein thrombus. Diagnoses for liver decompensations and other liver complications were identified based on *International Statistical Classification of Diseases and Related Health Problems, Tenth Revision,* codes (full list of codes in the eAppendix in Supplement 1).

### Statistical Analysis

Statistical analysis was performed from January 2023 to December 2025. Univariate analyses were conducted using *t* tests and χ^2^ tests to assess associations between MAUDs and sociodemographic factors or clinical covariates. Trends in mortality risk were calculated on the basis of number of MAUDs and by duration of MAUD use. Kaplan-Meier survival curves and log-rank tests were used to assess associations with MAUDs and survival. To control for confounding, we used multivariable Cox proportional hazards regression modeling with a tiered approach to adjust for sociodemographic and clinical covariates. We confirmed that the proportional hazards assumption was not violated (*P* = .11) and used a variance inflation factor cutoff of less than 3 to address collinearity. Given the importance of liver transplant status in mortality among this patient population, we conducted stratified analyses to examine mortality among those who never underwent liver transplantation. To control for confounding by indication for MAUD use, we conducted propensity score analysis using inverse probability weights to control for sociodemographic and clinical factors that may be associated with prescription of MAUDs. Further details are provided in eMethods 2 and eTable 1 in Supplement 1. All statistical analyses were conducted using R, version 4.3.1 (R Project for Statistical Computing). All *P* values were from 2-sided tests and results were deemed statistically significant at *P* < .05.

## Results

We identified 1309 patients (mean [SD] age, 57.1 [10.5] years; 989 men [75.6%] and 320 women [24.4%]; 35 Asian [2.7%], 41 Black [3.1%], 596 Hispanic [45.5%], 340 White [26.0%], 194 unknown race or ethnicity [14.8%], and 98 other race or ethnicity [7.5%]) who were evaluated by the transplant committee to have ALD as the primary cause of liver disease (Table 1). Patients had a mean (SD) follow-up of 38 (25) months, with a mean (SD) MELD-Na score of 22.6 (10.5) and a mean (SD) of 1.6 (0.7) hepatic decompensations. Only 467 patients (35.7%) were taking any form of AUD pharmacotherapy. Compared with those without MAUDs, the AUD pharmacotherapy group, on average, had a higher mean (SD) MELD-Na score (25.0 [10.8] vs 21.3 [10.1]; *P* < .001), higher mean (SD) Charlson Comorbidity Index (8.4 [3.3] vs 6.9 [3.1]; *P* < .001), and more hepatic decompensations (bleeding varices: 19.9% [93 of 467] vs 12.7% [107 of 842]; *P* < .001; hepatic encephalopathy: 53.5% [250 of 467] vs 40.4% [340 of 842]; *P* < .001).

**Table 1.** Cohort Characteristics by AUD Pharmacotherapy Usage

Characteristic	Any AUD pharmacotherapy (n = 467)	No AUD pharmacotherapy (n = 842)	Overall (N = 1309)	*P* value
Age, mean (SD), y	56.1 (10.6)	57.6 (10.5)	57.1 (10.5)	.01
Sex, No. (%)				
Female	130 (27.8)	190 (22.6)	320 (24.4)	.04
Male	337 (72.2)	652 (77.4)	989 (75.6)	
Race and ethnicity, No. (%)				
Asian, non-Hispanic	12 (2.6)	23 (2.7)	35 (2.7)	<.001
Black, non-Hispanic	16 (3.4)	25 (3.0)	41 (3.1)	
Hispanic	220 (47.1)	376 (44.7)	596 (45.5)	
White, non-Hispanic	136 (29.1)	204 (24.2)	340 (26.0)	
Unknown, non-Hispanic	36 (7.7)	158 (18.8)	194 (14.8)	
Other, non-Hispanic^a^	44 (9.4)	54 (6.4)	98 (7.5)	
ADI score, mean (SD)^b^	20.2 (17.0)	20.7 (17.1)	20.5 (17.0)	.67
MELD-Na score, mean (SD)	25.0 (10.8)	21.3 (10.1)	22.6 (10.5)	<.001
No. of decompensations, mean (SD)	1.7 (0.7)	1.5 (0.6)	1.6 (0.7)	<.001
Ascites, No. (%)	460 (98.5)	826 (98.1)	1286 (98.2)	.76
Bleeding varices, No. (%)	93 (19.9)	107 (12.7)	200 (15.3)	<.001
Hepatic encephalopathy, No. (%)	250 (53.5)	340 (40.4)	590 (45.1)	<.001
Hepatocellular carcinoma, No. (%)	111 (23.8)	206 (24.5)	317 (24.2)	.83
Charlson Comorbidity Index, mean (SD)	8.4 (3.3)	6.9 (3.1)	7.4 (3.2)	<.001

Abbreviations: ADI, Area Deprivation Index; AUD, alcohol use disorder; MELD-Na, Model for End-Stage Liver Disease–Sodium.

^a^ Included Indigenous, Middle Eastern or North African, multiple races, or self-identified as “other.”

^b^ The ADI is a zip code–based measure of neighborhood disadvantage that is linked with socioeconomic status, chronic illness, and early mortality. Higher scores suggest greater disadvantage.

Use of MAUDs was associated with progressively lower risk of all-cause mortality with an increasing number of MAUDs: among those with no MAUDs, 289 of 842 (34.3%) died, compared with 1 of 10 patients (10.0%) with 3 MAUDs (*P* = .005) (Table 2). Increasing duration of MAUDs was also associated with progressively lower risk of all-cause mortality: among those with no MAUDs, 312 of 900 (34.7%) died, compared with 12 of 95 patients (12.6%) with more than 6 months of MAUDs (*P* < .001) (Table 3). Compared with those not taking any AUD pharmacotherapy, the absolute risk reduction of death was 3% among those with 1 medication for AUD (number needed to treat [NNT] = 31) and 8% among those with 2 medications for AUD (NNT = 12) (Table 2). These associations persisted independently of MELD-Na score, liver transplant status, liver complications, medical comorbidities, and sociodemographic factors, with lower risk of all-cause mortality among those with at least 3 months of MAUDs compared with those with less than 3 months of MAUDs (hazard ratio [HR], 0.56; 95% CI, 0.36-0.89) (Table 4; Figure). These mortality associations also remained statistically significant after adjusting for propensity scores for use of MAUDs (HR, 0.59; 95% CI, 0.39-0.92) (Table 4). Use of MAUDs for at least 3 months was associated with 6.6% higher 1-year survival (SE, 0.02%) and 18.5% higher 3-year survival (SE, 0.03%).

**Table 2.** Trends in Mortality by AUD Pharmacotherapy Use Among Patients With Severe Alcohol-Related Liver Disease^a^

No. of AUD medications taken	Overall No.	No. (%)		NNT
		Deceased	Not deceased	
0	842	289 (34.3)	553 (65.7)	NA
1	380	118 (31.1)	262 (68.9)	31
2	77	20 (26.0)	57 (74.0)	12
3	10	1 (10.0)	9 (90.0)	4

Abbreviations: AUD, alcohol use disorder; NA, not applicable; NNT, number needed to treat to prevent 1 death compared with taking 0 AUD medications.

^a^ *P* = .005 for mortality trend.

**Table 3.** Trends in Mortality by AUD Pharmacotherapy Duration Among Patients With Severe Alcohol-Related Liver Disease^a^

Duration of AUD pharmacotherapy, mo	Overall No.^b^	No. (%)	
		Deceased	Not deceased
0	900	312 (34.7)	588 (65.3)
<3	178	62 (34.8)	116 (65.2)
3-6	53	15 (28.3)	38 (71.7)
>6	95	12 (12.6)	83 (87.4)

Abbreviation: AUD, alcohol use disorder.

^a^ *P* < .001 for mortality trend.

^b^ Sample size reflects availability of pharmacotherapy duration data.

**Table 4.** Adjusted Associations Between AUD Pharmacotherapy and Survival Among Patients With Severe Alcohol-Related Liver Disease

Model No.	Model parameters	All-cause mortality, hazard ratio (95% CI)	
		<3 mo AUD pharmacotherapy	≥3 mo AUD pharmacotherapy
1	Crude association	1 [Reference]	0.41 (0.28-0.61)
2	Adjusts for MELD-Na and liver transplant status	1 [Reference]	0.56 (0.38-0.83)
3	Adjusts for model 2 and decompensations, liver complications, and Charlson Comorbidity Index^a^	1 [Reference]	0.58 (0.39-0.86)
4	Adjusts for model 3 and age, sex, race and ethnicity, language, marital status, and ADI score^b^	1 [Reference]	0.56 (0.36-0.89)
5	Adjusts for model 4 and AUD pharmacotherapy propensity scores	1 [Reference]	0.59 (0.39-0.92)

Abbreviations: ADI, Area Deprivation Index; AUD, alcohol use disorder; MELD-Na, Model for End-Stage Liver Disease–Sodium.

^a^ Decompensations include bleeding varices, ascites, and hepatic encephalopathy. Liver complications include hepatocellular carcinoma, hepatorenal syndrome, hepatopulmonary syndrome, coagulopathy, and portal vein thrombus.

^b^ The ADI is a zip code–based measure of neighborhood disadvantage that is linked with socioeconomic status, chronic illness, and early mortality.

**Figure.**
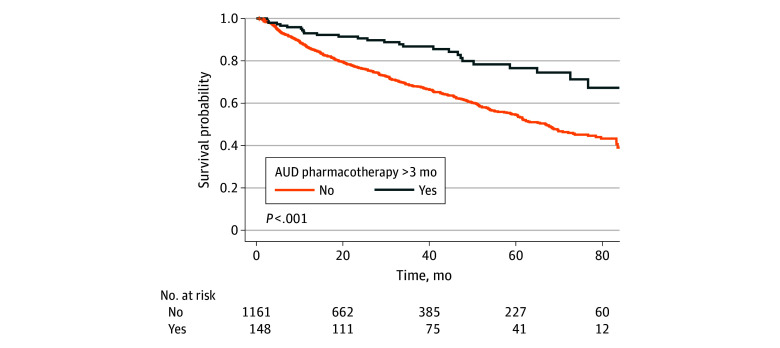
Kaplan-Meier Curve for Association Between Alcohol Use Disorder (AUD) Pharmacotherapy and Survival Among Patients With Severe Alcohol-Related Liver Disease

During the study, 327 patients underwent liver transplantation for ALD. Compared with patients without any MAUDs, those with any MAUDs were more likely to receive a liver transplant (39.2% [183 of 467] vs 17.2% [145 of 842]; *P* < .001), with an even higher likelihood of liver transplant among those with at least 3 months of MAUDs (48.6% [72 of 148]). Associations between MAUDs and lower risk of all-cause mortality persisted in subgroup analyses among those who did not undergo liver transplantation (HR, 0.53; 95% CI, 0.31-0.91) (eTable 2 in Supplement 1).

## Discussion

In this single-center cohort study of patients referred for liver transplantation for ALD as the primary indication, pharmacotherapy for AUD was associated with lower all-cause mortality. Longer duration of use and higher number of different MAUDs were both associated with lower mortality rates. This study adds to the current literature linking MAUDs with lower mortality,^14,23^ but with benefit from a wider range of MAUDs shown over a longer follow-up period of up to 3 years. A key highlight in this study was lower mortality associated with use of MAUDs despite higher severity of illness within the MAUD group based on MELD-Na score, Charlson Comorbidity Index, and liver decompensations.

Prior studies have shown that treatment for AUD can decrease the incidence and progression of ALD,^13,14^ decrease rehospitalization, and improve short-term mortality,^14,23^ with the present study offering support for long-term mortality benefits even among patients with multiple decompensations who are referred for liver transplant. Furthermore, systematic studies have shown that MAUDs are associated with a low NNT to achieve their benefit with alcohol cessation, with our study similarly associating a low NNT with mortality prevention.^24^

However, AUD remains a complex disease with overall low treatment rates.^15^ Effective AUD treatment often addresses several underlying driving factors for AUD, such as prior history of trauma, complex relational trauma, and concomitant mental health disorders, alongside social determinants of health. Such treatment plans require a nuanced approach by combining MAUDs with psychosocial interventions individualized to each patient. However, multifaceted barriers to AUD treatment exist. Patients with AUD face stigma and financial barriers to seeking clinical addiction services, which are also of limited availability overall. Clinicians with less experience may not adequately screen for AUD and may not be familiar with initiating pharmacotherapy for AUD, especially for patients with severe ALD. Limited research is available regarding the association of MAUDs with the psychosocial dynamics of ALD beyond reduction in alcohol consumption that may potentially play a role in disease modification. In this study, the overall rates of MAUD use were higher compared with prior published estimates, but it is unclear if combination therapies seen in the present study may have offered unexplained benefits for the psychosocial components of ALD beyond alcohol reduction, a topic that requires further study. Although the current study is unable to address these factors when considering treatment with MAUDs, the propensity score analysis shows an association between treatment with MAUDs and long-term survival among this population with severe ALD. These data support clinical and policy interventions to address barriers to AUD treatment, especially among patients with severe ALD referred for liver transplant. Recent advocacy has suggested a new model of multidisciplinary treatment approach with colocated hepatology and addiction services to improve access and outcomes for both AUD and ALD treatment.^25^

### Limitations

The present study has some limitations. As a single-center study, these findings may have limited generalizability beyond similar tertiary referral centers. Inferences regarding the causality of this association are limited by the observational nature of these data, which are also susceptible to immortal time bias. The overall trend with lower mortality is, however, supported by the magnitude, plausibility, and dose response of these associations by number and duration of MAUDs, in addition to persistence in multivariable regression modeling to control for several measured confounders.

Although use of MAUDs could be susceptible to a selection bias for those who are healthier overall, as noted in the inverse association with MAUD prescription and cirrhosis decompensation in the previous study showing mortality benefit,^14^ our cohort characteristics reflect the contrary, with patients within the MAUD group in our study having higher MELD-Na scores, more comorbidities, and more liver decompensations.

The study findings are susceptible to measurement error. With regard to the study outcome, our EMR data are overall robust regarding mortality status for patients referred for transplant who are generally followed up closely. However, these measures are still subject to loss to follow-up. Future studies can explore cross-validation with government vital records.

Another limitation of the study is that we do not have accurate measures to control for use of mental health care and nonpharmacologic treatments for AUD, and their potential association with alcohol use and mortality independent of MAUDs. In addition, our data are blinded to the indication of each prescribed treatment. To mitigate the associations of these considerations we used propensity score adjustment to control for confounding by indication based on a wide range of sociodemographic and clinical factors, including mental health diagnoses.

Last, the final 2 years of our study period overlap with the COVID-19 pandemic. Although the first 4 years of our study period predate the pandemic and any related impact, it is possible the increase in ALD during and since the pandemic imposes a temporal trend in our study population. Further studies will be needed to corroborate these findings in a fully post–COVID-19 cohort.

Ultimately, the findings in our study will need corroboration and replication in further studies to demonstrate generalizability and explore mediation via measures of alcohol intake. Future studies using a randomized intervention or quasi-experimental design can further address additional potential biases in these findings.

## Conclusions

AUD remains a complex disorder, with effective treatments but low use of treatments. This retrospective single-center cohort study found that pharmacotherapy for AUD was associated with improved 1- and 3-year survival among patients with severe ALD referred for liver transplantation. These findings support the need to further improve access to AUD treatment among patients with severe ALD.

## Notes

Supplement 1. **eAppendix.** *ICD* Codes **eMethods 1.** Text Parsing Details **eFigure.** CONSORT Diagram of the Sample Selection **eMethods 2.** Propensity Score Matching **eTable 1.** Propensity Score Diagnostics **eTable 2.** Adjusted Associations Between AUD Pharmacotherapy and Survival Among Patients With Severe ALD Who Did Not Receive Liver Transplant
eReference.

Supplement 2.
Data Sharing Statement
