# Supplementary material for: Medications for Alcohol Use Disorder Among Patients With Severe Alcohol-Related Liver Disease
Source: JAMA Netw Open. 2026 Feb 11;9(2):e2559016. doi: 10.1001/jamanetworkopen.2025.59016 (PMC12895287; doi:10.1001/jamanetworkopen.2025.59016)
Supplement: Supplement 1. — eAppendix. ICD Codes eMethods 1. Text Parsing Details eFigure. CONSORT Diagram of the Sample Selection eMethods 2. Propensity Score Matching eTable 1. Propensity Score Diagnostics eTable 2. Adjusted Associations Between AUD Pharmacotherapy and Survival Among Patients With Severe ALD Who Did Not Receive Liver Transplant eReference. [file jamanetwopen-e2559016-s001.pdf]

Supplementary Online Content

Sundaresh R, Singh J, Meza J, Saab S, Shetty A. Medications for alcohol use disorder among patients with severe alcohol-related liver disease. *JAMA Netw Open.* 2026;9(2):e2559016. doi:10.1001/jamanetworkopen.2025.59016

eAppendix. *ICD* Codes

eMethods 1. Text Parsing Details

eFigure. CONSORT Diagram of the Sample Selection

eMethods 2. Propensity Score Matching

eTable 1. Propensity Score Diagnostics

eTable 2. Adjusted Associations Between AUD Pharmacotherapy and Survival Among Patients With Severe ALD Who Did Not Receive Liver Transplant

eReference.

This supplementary material has been provided by the authors to give readers additional information about their work.

eAppendix. *ICD* Codes

The following ICD codes were used to categorize clinical covariates. These codes were derived from local practice patterns for clinical coding at our tertiary center.

Alcohol-related liver disease: K70, K70.0, K70.1, K70.10, K70.11, K70.2, K70.3, K70.30, K70.31, K70.4, K70.40, K70.41, K70.9

Decompensations

Ascites: R18, R18.0, R18.8, K70.31, K70.11, K71.51

Bleeding varices: I85.01, I85.11

Hepatic encephalopathy: K76.82

Other liver-related complications

Spontaneous bacterial peritonitis: K65, K65.0, K65.2, K65.3, K65.9, K65.9

Varices: I85, I85.0, I85.00, I85.01, I85.10, K85.11, I86.4

Hepatorenal syndrome: K76.7

Coagulopathy: D68.4, D68.8, D68.9

Hepatopulmonary syndrome: K76.81

Hepatocellular carcinoma: C22.0

Portal vein thrombus: I81

Other

Uncomplicated AUD: F10.10, F10.120, F10.130, F10.20, F10.220, F10.230, F10.90, F10.920

Mood disorders: F30, F31, F32, F33, F34, F39

Psychotic disorders: F20, F21, F22, F23, F25, F28, F29

© 2026 Sundaresh R et al. *JAMA Network Open.*

eMethods 1. Text Parsing Details

We used systematic text parsing to identify patients with ALD as the primary etiology of liver disease. First, we filtered clinic notes by note type, selecting for transplant committee review notes. Next, we filtered for committee review notes that were specific for liver transplant (i.e. excluding notes from heart transplant committee meetings, or for other organs). Transplant committee notes have a standardized note template which includes a section for primary diagnosis. This section of the note was selected for with additional handling of extraneous leading or trailing characters.

Finally, patients were coded according to the listed primary diagnosis of liver disease as determined by the liver transplant committee. For ALD this was variously documented as “ETOH”, “Alcohol”, or “Laennec” by the transplant committee. These patients were all coded as having ALD as the primary etiology of liver disease and were included in our study. Similarly, based on the transplant committee notes, patients were coded as having alternate primary etiologies of liver disease including: MASH (formerly NASH), HCV, HBV, PBC, PSC, Autoimmune hepatitis, A1AT deficiency, acute cellular rejection, graft failure, chronic rejection, congestive hepatopathy, Budd-Chiari syndrome, cholangiocarcinoma, metastatic neuroendocrine tumor, polycystic liver disease, congenital liver disease, Caroli’s disease, or cryptogenic liver disease. Patients who had a primary etiology of liver disease other than ALD were not included in our study.

© 2026 Sundaresh R et al. *JAMA Network Open.*

eFigure. CONSORT Diagram of the Sample Selection

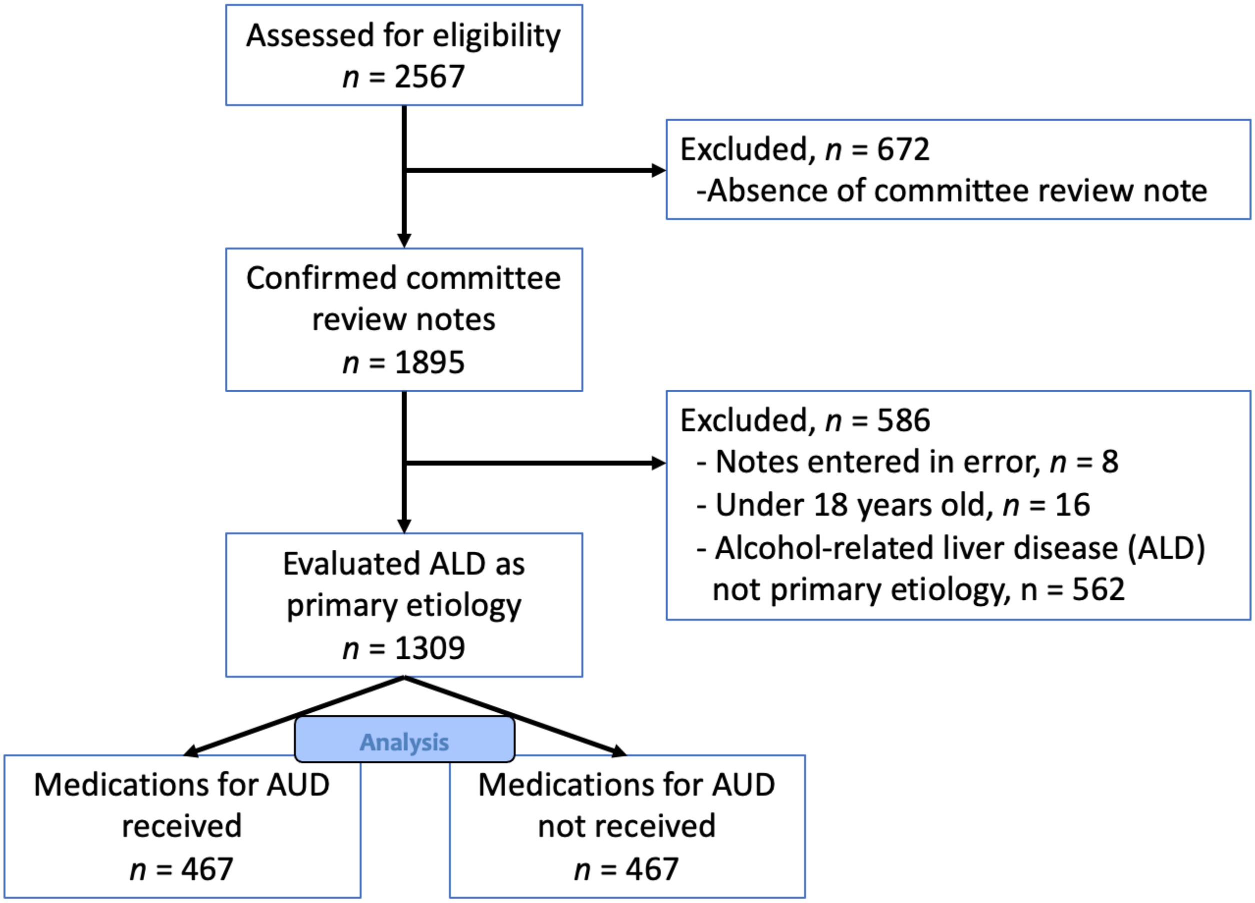

- 2567 total patients referred for liver transplant evaluation for ALD as etiology during the study period
  - 1895 patients who completed a transplant committee evaluation with a documented committee review note during the study period
    - 8 notes were entered in error
    - 16 patients were <18 years old
    - 562 patients had etiologies other than alcohol as the primary etiology of liver disease (as determined by the transplant committee)
- Included in analysis: 1309 patients with ALD as the primary etiology of liver disease
  - 467 patients with any AUD pharmacotherapy
  - 842 patients without AUD pharmacotherapy

© 2026 Sundaresh R et al. *JAMA Network Open.*

eMethods 2. Propensity Score Matching

In order to control for confounding by indication of mAUD usage, we conducted a propensity score analysis. First we built a logistic regression model to estimate the predicted probability of using any mAUD for at least 3 months. Covariates in this model included MELD, age, sex, race/ethnicity, language, marital status, ADI score, mood disorders, psychotic disorders, liver decompensations, other liver-related complications, uncomplicated AUD, liver transplant status, and Charleston comorbidity score. Next, propensity wights were calculated using the inverse probability treatment weighting method.[1] For patients who did not use mAUD for at least 3 months, inverse probability was calculated as $\frac{1}{1- predicted\ probability}$, and for those who did use mAUD for at least 3 months, inverse probability was calculated as $\frac{1}{predicted\ probability}$. These propensity weights were then incorporated into a weighted cox proportional hazards model of the main analysis.

© 2026 Sundaresh R et al. *JAMA Network Open.*

eTable 1. Propensity Score Diagnostics

Variable	Standardized Mean Difference
Any mAUD >= 3 months	-0.15
MELD	0.54
Liver transplant	-0.25
Decompensations	0.17
Liver complications (including hepatocellular carcinoma, hepatorenal syndrome, hepatopulmonary syndrome, coagulopathy, portal vein thrombus)	0.12
Charleson comorbidity index	0.22
Age	0.03
Sex— Male	0.008
Race/ethnicity— Asian (non-Hispanic)	<0.001
Race/ethnicity— Black (non-Hispanic)	-0.03
Race/ethnicity— Hispanic	-0.02
Race/ethnicity— Other (non-Hispanic)	-0.02
Race/ethnicity— Unknown (non-Hispanic)	0.05
Race/ethnicity— White (non-Hispanic)	0.02
Language— English	-0.06
Language— Other	0.004

© 2026 Sundaresh R et al. *JAMA Network Open.*

Language— Spanish	0.05
Marital status— Married or significant other	-0.10
Marital status— Single	0.02
Marital status— unknown	0.08
Marital status—widowed, divorced, or separated	-0.01
ADI score	-0.02

Results

eTable 2. Adjusted Associations Between AUD Pharmacotherapy and Survival Among Patients With Severe ALD Who Did Not Receive Liver Transplant

		**All-cause mortality, HR (92% CI)**	
**Model**	**Model Parameters**	**<3 months AUD pharmacotherapy**	**>3 months AUD pharmacotherapy**
Model 1	Crude association	1 [Reference]	0.55 (0.35-0.87)
Model 2	Adjusts for MELD	1 [Reference]	0.57 (0.36-0.90)
Model 3	Adjusts for Model 2 and decompensations, liver complications, and Charlson comorbidity index	1 [Reference]	0.60 (0.38-0.95)
Model 4	Adjusts for Model 3 and age, sex, race/ethnicity, language, marital status, and ADI score	1 [Reference]	0.53 (0.31-0.91)

AUD = alcohol use disorder

MELD = Model for End-stage Liver Disease

Decompensations include bleeding varices, ascites, and hepatic encephalopathy

Liver complications include hepatocellular carcinoma, hepatorenal syndrome, hepatopulmonary syndrome, coagulopathy, and portal vein thrombus

ADI = Area Deprivation Index, a zipcode-based measure of neighborhood disadvantage that is linked to socioeconomic status, chronic illness, and early mortality

Restricted Mean Survival Time:

5-year RMST: 4.4 years (amongst those with AUD pharmacotherapy for at least 3 months) vs 3.7 years (amongst those without AUD pharmacotherapy for at least 3 months), P<0.001.

© 2026 Sundaresh R et al. *JAMA Network Open.*

eReference.

1. Chesnaye NC, Stel VS, Tripepi G, Dekker FW, Fu EL, Zoccali C, Jager KJ. An introduction to inverse probability of treatment weighting in observational research. Clin Kidney J. 2021 Aug 26;15(1):14-20. doi: 10.1093/ckj/sfab158.

© 2026 Sundaresh R et al. *JAMA Network Open.*
