# Supplementary material for: Medications for Alcohol Use Disorder Among Patients With Severe Alcohol-Related Liver Disease
Source: JAMA Netw Open. 2026 Feb 11;9(2):e2559016. doi: 10.1001/jamanetworkopen.2025.59016 (PMC12895287; doi:10.1001/jamanetworkopen.2025.59016)
Supplement: Supplement 2. — Data Sharing Statement [file jamanetwopen-e2559016-s002.pdf]

Data Sharing Statement

Sundaresh. Medications for Alcohol Use Disorder Among Patients With Severe Alcohol-Related Liver Disease. *JAMA Netw Open*. Published February 11, 2026. doi:10.1001/jamanetworkopen.2025.59016

Data
Data available: No

Additional Information
Explanation for why data not available: data will be made available if requested based on IRB permission from institution, but currently not included in the manuscript
